# Prevalence of primary headache disorders among information technology staff in China: the negative effects of computer use and other correlative factors

**DOI:** 10.1186/s12889-020-08497-9

**Published:** 2020-04-05

**Authors:** Chunlin Li, Lei Zhang, Jin Zhou, Zhiliang Fan, Yan Wang, Xiaolin Wang, Weidong Wang, Shengyuan Yu

**Affiliations:** 1 Department of Neurology, Chinese PLA General Hospital, 28 Fuxing Road, Haidian District, Beijing, 100853 China; 2 The third department of Neurology, Affiliated Xing Tai People’s Hospital of Hebei, Medical University, Xingtai, 054000 Hebei Province China; 3 Center of Medical Device and Clinical Evaluation, Chinese PLA General Hospital, 28 Fuxing Road, Haidian District, Beijing, 100853 China

**Keywords:** Primary headache, Prevalence, Computer use, Information technology

## Abstract

**Background:**

To date, there have been very few studies that have explored the relationship between headaches and computer use. The chief aim of this study is to investigate the prevalence of primary headache disorders among informational technology staff and identify the potential factors contributing to it.

**Methods:**

This is a cross-sectional study based on annual health checks of employees from the information technology industry. We identified 2216 information technology staff members from Beijing by stratified random sampling who met the inclusion criteria. All participants were initially required to have a physical examination, after which they complete a general situation questionnaire that included a headache screening question. Those who had suffered from headache within the previous year also completed the questionnaire developed by Lifting the Burden. The International Classification of Headache Disorders 3(ICHD-3) criteria was used for the diagnosis of headache.

**Results:**

A total of 2012 valid questionnaires (males, 1544 [76.7%]; females, 468 [23.3%]) were obtained from 2216 participants for a response rate of 90.8%. A total of 619 participants were diagnosed with primary headache, the one-year prevalence of which was 30.8%. Regarding the classification of the primary headache, 152 participants suffered from migraine, with a one-year prevalence of 7.6%; 440 and 27 suffered from tension-type headache and unclassified headaches, with one-year prevalences of 21.9 and 1.3%, respectively. Multivariate regression analysis showed that female gender was a risk factor for migraine and tension-type headache (OR 3.21[95% CI 2.28–4.53] and 1.88[95% CI 1.47–2.40], respectively). Age was also related to migraine and tension-type headache. The 41–50 age group had 2.02 times the probability of migraine, and the 31–40 age group had 1.89 times the probability of tension-type headaches compared to the 18–30 age group. Obesity and excessive computer use (more than 12 h per day) were also factors contributing to tension-type headache (OR: 2.61[95% CI 1.91–3.56] and 1.63[95% CI 1.18–2.25], respectively).

**Conclusions:**

The one-year prevalence of primary headache in this population was 30.8%. The prevalence of tension-type headache in this population was higher than that in the general Chinese population. The occurrence of primary headache is correlated with many factors, among which excessive computer use significantly contributed to the risk of tension-type headache.

## Background

Primary headaches, especially migraine and tension-type headaches (TTH), are among the most common and most costly diseases in the world [1]. The prevalence of primary headache has been insufficiently investigated in China and other developing countries. According to statistics, the global 1-year prevalence of primary headache in adults is 47, 10% of which are migraine, while tension-type headache accounts for 35% [2]. Nearly all the patients with migraine and approximately half of the patients with TTH had at least one headache attack that affected their daily activities. Characterized by severe paroxysmal unilateral pulsatile headaches and often accompanied by nausea, vomiting, photophobia, phonophobia and other symptoms, migraine has been the most thoroughly studied type of primary headache. The effects of migraine on quality of life and the ability to work are noticeable, and it was identified as the seventh most disabling disease in the Global Burden of Disease Survey 2010 by the World Health Organization [3]. Compared with migraine, studies on TTH have been rare. However, TTH is more commonly seen in headache clinics, and the prevalence rate of TTH is much higher than that of migraine. Various studies have shown that the lifetime prevalence rate of TTH among the population ranges from 30 to 78%. TTH has a huge impact on social economics, and its repeated attacks also affect patients’ quality of life, which is often further complicated by insomnia and psychological disorders [4, 5]. In China, a study based on the national population showed that the 1-year prevalence of primary headache in the 18–65 age group was 23.8% [6]. Primary headache is underestimated, underdiagnosed and undertreated in China and many other developing countries [7]. Social, economic and educational status can all affect the individual experiences of headache sufferers, especially in developing countries. For example, triptans are among the most common used medications in many developed countries but in China few patients suffering from primary headache know about triptans.

Due to the higher prevalence and the consequent decline in the ability to work, the prevalence of headaches in particular occupational groups is worthy of our attention. With the development of the internet, information and internet technology (IT) have rapidly progressed in China, affecting a wide portion of the population and bringing profound changes to the daily life of adults [8, 9]. The information and internet technology industry has become one of the busiest fields currently in China. Excessive computer use is associated with both tension-type headache and migraine [10–12]. And computer use is especially common among IT staff who use computers for working, studying and playing. In this context, information technology staffs often face many health problems, including headache. To the best of our knowledge, no studies have investigated the prevalence of headache among information technology staff in China. Therefore, a need to identify factors associated with the prevalence of headache among this population has arisen. Our study aims to estimate the prevalence of primary headaches and explore the factors correlated with these headache disorders among information technology staff in China. In addition, we assessed the negative influence of several occupational factors on headache disorder. Based on this study, some corresponding intervention measures are suggested that might improve the health status quality of life of the IT population to further advance their work efficiency and social and economic benefits.

## Methods

### Sampling method

Multistage stratified systematic random sampling was used to select the participants from among the staff in information technology in Beijing. We selected and engaged with participants from large internet companies that spread over 10 major Beijing districts (Xicheng, Dongcheng, Haidian, Chaoyang, Fengtai, Shijingshan, Changping, Fangshan, Tongzhou, Huairou). Based on the number of social security payers in company, we identified two hundred internet companies with 200–500 employees, and arranged these companies in alphabetical order. The first of every twentieth companies in the list was asked to participate. When a company refused to participate in the survey, we recruited from the next company in the sequence. Based on the reported prevalence of headache (approximately 50% and the absolute margin of error of 2% with 95% confidence interval), a minimum of 1900 subjects was needed. To overcome the limitation of invalid data, we expanded the sample size by a further 10%.

### Questionnaire and survey

Data collection was completed via a questionnaire survey over 5 months, from March to July 2018, in Beijing. All subjects were recruited during annualhealth checksto ensure that valid responses to our questions were obtained. The questionnaire consisted of two sections: basic sociodemographic variables and headache characteristics. All participants were asked to fill in the sociodemographic section including age, gender, body mass index, educational attainment, occupation factors and so on. At the end of the sociodemographic section, a screening question for headache (Did you have any headache attack over the previous year?) was addressed to all respondents. The participants were identified as headache-free if their answers were “no”. Only those who answered “yes” were asked to subsequently fill out the headache characteristics section. Headache characteristics included the following items: (1) pain site, (2) pain type (nature), (3) attack duration, (4) associated symptoms, (5) headache days/month (in last 3 months), and (6) visual analog scale (VAS) score.

Face-to-face headache interviews were conducted in the office buildings of the IT staff by five professional neurologists with participants who reported a history of headache. The participants who were out of the city during the survey were asked to perform a telephone interview to guarantee the participation rate. The questionnaire and interview guide used in our study have previously been adopted in our team’s previous research [6].

### Diagnosis and data analysis

The participants who reported headaches received a detailed examination regarding the headache characteristics (pulsating, aching, duration, location, intensity, frequency, accompanying symptoms, etc.). Primary headaches were classified into migraine, tension-type headache (TTH) and unclassified primary headache. Migraine and TTH were diagnosed based on ICHD-III criteria, with differences examined by other neurologists. To arrive at a diagnosis in response to these differences, the ICHD-III criteria were applied in the following order: migraine, TTH, probable migraine, probable TTH. If the standard criteria were not met, the participants were diagnosed with unclassified headache. The respondents who might have more than one type of headache were instructed to focus on the most bothersome type, which means that only one headache type was diagnosed. All five neurologists involved were trained together to ensure a consistent diagnosis. Cases of definite and probable migraine or TTH were combined for prevalence estimation and further analysis. Secondary headaches caused by tumor, trauma and infection were not included in these analyses.

### Statistics

Statistical analyses were performed by using Statistical Package for Social Science 16.0. Normally distributed data were expressed as the means±standard deviations (SD); categorical data were summarized as the number and percentages (%). In the single factor analysis, the measurement data were analyzed by one-way analysis of variance (ANOVA), and the LSD-t method was used to compare data between groups. To order multicategorical variables, enumeration data were assessed by the Kruskal-Wallis H test. We used the type of headache as the dependent variable in the multivariate analysis to evaluate different factors associated with headache by using logistic regression analysis. We calculated 95% confidence intervals (CIs) and 95% odds ratios (ORs) of risk factors for migraine and TTH. Statistical significance was set at *P* < 0.05.

## Results

A total of 2216 subjects from nine companies were enrolled in the study, all of whom were required to finish a semistructured questionnaire. Most of the subjects were middle-aged and came from all over China. Among the 2216 participants, 116 were absent from the survey because of business or vacation, 88 submitted incomplete or unreliable questionnaires, and 2012 completed the survey correctly and effectively. The overall response rate was 90.8%. The participants in the study were aged from 18 to 60 years (mean 36.0 ± 9.2 years), and most of them were male (76.7%), well educated (master’s or higher degree,45.5%) and married (69.6%). More female participants tended to be single and have less work experience compared to males (Table 1).

**Table 1:** Socio-demographic characteristics and 1-year prevalence of headaches classified by gender

	Total (*n* = 2012,%)	Men (*n* = 1544, %)	Women (*n* = 468, %)
**Age(years)**			
18–30	702 (34.9)	526 (34.1)	176 (37.6)
31–40	718 (35.7)	549 (35.6)	169 (36.1)
41–50	385 (19.1)	293 (19.0)	92 (19.7)
51–60	207 (10.3)	176 (11.3)	31 (6.6)
**Mean age (SD) years**	36.0 ± 9.2	36.1 ± 8.3	35.8 ± 9.0
**Education**			
Bachelor or lower	1097 (54.5)	830 (53.8)	267 (57.1)
Master or higher	915 (45.5)	714 (46.2)	201 (42.9)
**Marriage**			
single	611 (30.4)	436 (28.2)	175 (37.4)
married	1401 (69.6)	1108 (71.8)	293 (62.6)
**Work experience (years)**			
1–5	888 (44.1)	664 (43.0)	224 (47.9)
6–10	681 (33.8)	514 (33.3)	167 (35.7)
10-	443 (22.1)	366 (23.7)	77 (16.4)
**Headache, all types**	619 (30.8)	396 (25.6)	223 (47.7)
**Migraine**	152 (7.6)	81 (5.2)	71 (15.2)
Migraine without aura	107 (5.3)	48 (3.1)	59 (12.6)
Migraine with aura	17 (0.8)	13 (0.8)	4 (0.9)
Probable migraine	28 (1.4)	20 (1.3)	8 (1.7)
Chronic migraine	0	0	0
**TTH**	440 (21.9)	297 (19.2)	143 (30.6)
infrequent episodic TTH	233 (11.6)	156 (10.1)	77 (16.5)
frequent episodic TTH	117 (5.8)	82 (5.3)	35 (1.7)
Probable TTH	74 (3.7)	51 (3.3)	23 (4.9)
Chronic TTH	12 (0.6)	4 (0.2)	8 (0.4)
^**a**^**Unclassified headache**	27 (1.3)	18 (1.2)	9 (1.9)

^a^Unclassified headache include 3.1 cluster headache (7 cases), 3.2 Paroxysmal hemicranias (5 cases), 4.2 Primary exercise headache (5 cases), 4.5 Cold-stimulus headache (8 cases) and 4.8 Nummular headache (2 cases)

### Headache prevalence

Table 1 also displays the one-year prevalence of different types of primary headache in this population. Of the 2012 eligible participants, 619 experienced a history of primary headache in the preceding year (1-year prevalence 30.8%; males, 25.6%; females, 47.7%).

Among the 619 participants who had headaches, 152 (7.6%) were diagnosed with migraine, and 440 (21.9%) were diagnosed with TTH. The headaches were unclassifiable in 27 (1.3%) individuals. Due to professional particularities, young male staffs hold the dominant position in the IT industry, which does not match the gender distribution in the general population. Therefore, we displayed the prevalence of headache by gender. The one-year prevalence of migraine in males was 5.2%, while in females the proportion was higher at 15.2%. The one-year prevalence of TTH in males was 19.2%, while in females the proportion was higher at 30.6%.

### Prevalence in subgroups with different demographic characteristics

Table 2 provides the prevalence in subgroups with different demographic characteristics. Primary headache was more prevalent in females than in males for migraine and TTH (*P* < 0.001). In migraine patients, the 31- to 40-year-old group had the highest prevalence in both males and females, which decreased with aging in females but not in their male counterparts. The 1-year prevalence of TTH also peaked in the 31–40 year-old (Y/O) group and then declined with increase of age in both genders.

**Table 2:** The demographic characteristics comparisons between different types of headaches and non-headache participants

Variable	All headache (%)	Migraine (%)	TTH (%)	Unclassified headache (%)
**Gender**				
Male	396 (25.6)	81 (5.2)	297 (19.2)	18 (1.2)
Female	223 (47.7)	71 (15.2)	143 (30.6)	9 (1.9)
*P*	< 0.001	< 0.001	< 0.001	0.2123
**Age (years)**				
18–30	173 (24.6)	41 (5.8)	128 (18.2)	4 (0.5)
31–40	257 (35.8)	63 (8.8)	188 (26.2)	6 (0.8)
41–50	129 (33.5)	34 (8.8)	87 (17.9)	8 (2.0)
51–60	60 (29.0)	14 (6.8)	37 (17.9)	9 (4.3)
*P*	< 0.001	0.1347	0.0047	< 0.001
**Marital status**				
married	409 (29.3)	103 (5.2)	281 (20.1)	25 (1.8)
single	210 (34.4)	49 (8.0)	159 (26.1)	2 (0.3)
*P*	< 0.001	< 0.001	< 0.001	< 0.001
**BMI**				
< 24	158/724 (21.8)	46 (6.4)	110 (15.2)	2 (0.3)
25–28	287/874 (32.8)	69 (7.9)	205 (23.5)	13 (1.5)
> 28	174/414 (42.0)	37 (8.9)	125 (30.2)	12 (2.9)
*P*	< 0.001	0.0557	< 0.001	0.0002
**Education**				
Bachelor or lower	292/1097 (26.6)	71 (6.5)	206 (18.8)	15 (1.4)
Master or higher	327/915 (35.7)	81/915 (8.9)	234 (25.6)	12 (1.3)
*P*	< 0.001	0.0442	0.0002	0.9136
**Computer work**				
< 8 (hours)	105/408 (25.7)	29 (7.1)	69 (14.7)	7 (1.7)
8–12 (hours)	224/729 (30.7)	55 (7.5)	158 (21.7)	11 (1.5)
> 12 (hours)	290/875 (33.1)	68 (7.8)	213 (24.3)	9 (1.0)
*P*	0.0278	0.6842	0.0110	0.5398
**Job category**				
Software Engineer	241/776 (31.1)	63 (8.1)	169 (21.8)	9 (1.2)
Application Engineer	266/858 (31.0)	61 (7.1)	192 (22.4)	13 (1.5)
Managerial	112/378 (29.6)	28 (7.4)	79 (27.4)	5 (1.3)
*P*	0.8176	0.7377	0.8430	0.8229

TTH was more commonly seen among participants with higher education attainment (master’s degree or higher) than the counterparts (bachelor’s degree or lower) (25.6% vs 18.8%, *P* = 0.0002). Single IT staff members were more susceptible than married staff to migraine (8.0% vs 5.2%, *P* = 0.001, respectively) and TTH (26.1% vs20.1%, *P* < 0.001, respectively), but for unclassified headaches, the situation was the opposite (0.3%vs1.8%, *P* < 0.001, respectively). All types of headache were more common in the obese group. The univariate analysis showed that the prevalence of TTH significantly differed among different BMI groups (*P* < 0.001). The risk of migraine was slightly linked with BMI (*P* = 0.0557). Excessive computer use was significantly associated with TTH (*P* = 0.011). However, we found no evident association between headache type and job category.

### Characteristics of headache

In our survey, migraine was characterized as unilateral (50.7%, 77/152) and pulsatile (67.8%, 103/152). Over half of the migraine patients (83/152; 54.6%) reported less than one headache episode per month. Most of the headaches lasted for 4–72 h (135/152, 88.8%). In 152 migraine patients, 14.5% (22) had aura symptoms before episodes, while the remaining 85.5% (130) did not. Migraine attacks are often accompanied by symptoms other than head pain. This study indicated that photophobia was the most common symptom prevalent in migraine patients. Apart from that, aura was reported more often in males than in females (19.8% vs 8.5%, *P* = 0.0235, respectively). The majority of the migraine patients had a VAS score of 7.

TTHs were bilateral (46.8%, 206/440) and nonpulsatile (81.4%, 358/440). A total of 69.3% (305/440) of the patients had less than one headache episode per month. The most common TTH duration in this population was less than one hour (292/440, 66.4%). The median VAS pain score was 2 in males and 3 in females.

### Multivariable adjusted odds ratio (95% confidence interval) for migraine and TTH

We also used multivariate logistic regression analysis to inspect factors associated with migraine and TTH (Tables 3 and 4). This analysis confirmed that female sex, age 41–50 years and participants with higher education attainment were factors associated with migraine, and female sex, age 31–40 years, single status, BMI > 25 and computer use> 8 h were factors associated with TTH.. Neither migraine nor TTH was associated with job category.

**Table 3:**
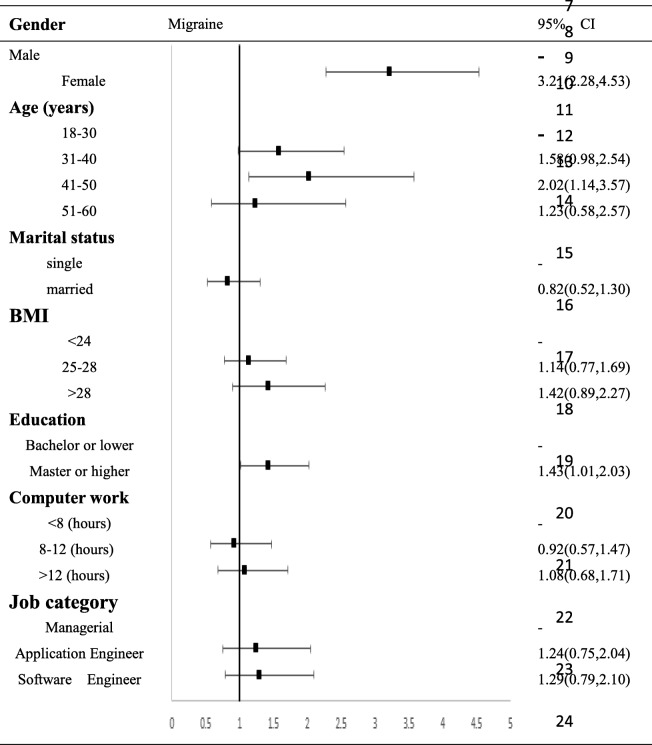
Multivariate logistic regression analyses of associations of migraine

**Table 4:**
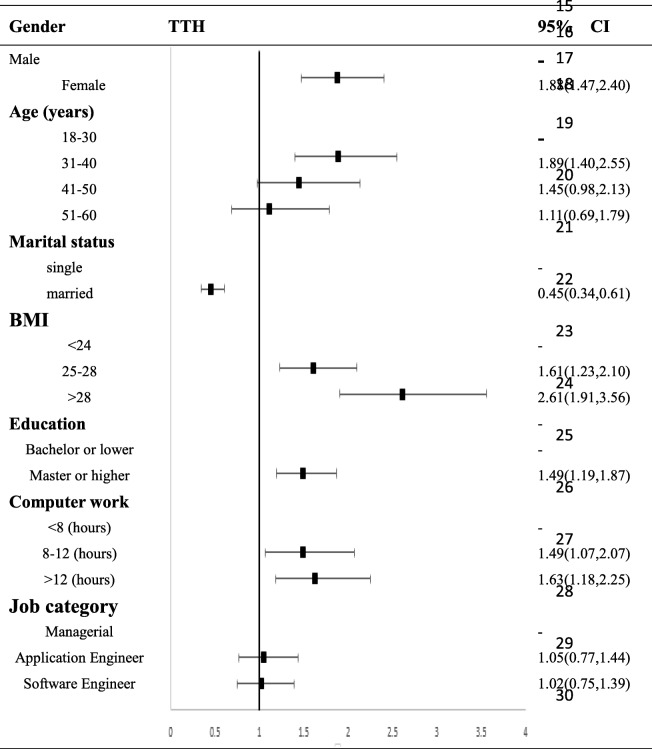
Multivariate logistic regression analyses of associations of TTH

## Discussion

The 1-year prevalence of primary headache in this population was 30.8% overall and 25.6% in males and 47.7% in females. Primary headache has been shown to be more prevalent in females than in males [6, 13–15]. Due to the occupational particularity of the IT profession, 76.7% of participants in our survey were male, which does not match the gender distribution in the general population. The prevalence of primary headache in this population should be lower than that in the general population because this study included a greater proportion of male participants. However, the prevalence of primary headache in this population was higher than that in the general population of Mainland China [6], which means that this group has a higher prevalence of headaches than the general population.

### Comparisons with studies in other countries and regions

For the purpose of making an honest statement of fact and statistical comparisons, we calculated the headache prevalence by gender and headache type. Our study observed a 1-year prevalence of 7.6%for migraine (5.2%for males and 15.2% for females) among the IT staff. Regional variability exists in the reported prevalence of migraine, with ranges of 1 to 22%in Asia, 9 to 16%in North America and 10 to 25% in Europe [16]. Our data on the prevalence of migraine were lower than those in the general population in mainland China (7.6% vs9.3%). The low prevalence of migraine in our study may have been due to the considerably higher proportion of the male population in the IT industry. After all, the predominance of women with migraine has been relatively consistent finding in many other studies [13]. In our study, 5.2% of the male participants reported migraine, while the proportion of migraine in the general male population in mainland China was 5.9%. The difference was not significant. However, the prevalence of migraine in the female participants was higher than that in the general female population in mainland China (15.2% vs 12.8%) [6], which was similar to some particular populations with high-intensity work, such as nurses or doctors [17–20]. A possible reason was that women may be more vulnerable to a combination of circumstances such as pressure and hormone. It is worth noting that migraine became more prevalent with advancing age until a peak was reached during the fifth decade of life. After that, it declined more quickly in women than in men. These findings were similar to those of previous studies [21–23].

TTH was the most common headache type in our survey, which confirms previous studies [1, 24–26], that showed a higher prevalence of TTH in the population. Regional variability also exists in the prevalence of TTH due to different participant characteristics or methodological variations. TTH was reported to be more prevalent in Europe than in other areas. The prevalence of TTH was previously estimated at 35–86%in European countries and 20–30% in Asia and America [27–29]. The 1-year prevalence of TTH in our study was21.9%, which appeared to be much higher than that in mainland China both in males (19.2% vs 7.7%, respectively) and in females (30.6% vs 14.0%, respectively). Many previous studies have suggested that intense stress at work is an associated factor for TTH [30–32]. IT staff usually work under huge pressure and intensity, which is probably the reason why the prevalence of tension-type headache was significantly higher than that in the general population. The prevalence of TTH in males and females peaked in mid-life and dropped to its lowest level in 51–60 years group in our study. These findings were similar to those reported in mainland China [29] and other Asian countries [27, 28]. Our prevalence estimate for TTH was still somewhat conservative. Two factors are relevant here. First, our study did not analyze coexistent migraine and TTH in the participants. Those with both migraine and TTH were likely to regard the former as the more bothersome, leading to a partial neglect of TTH. Second, the participants may not have considered infrequent TTH to be a health problem, so they tended to focus on the most bothersome TTH. Therefore, the prevalence of TTH in our study could have been somewhat conservative.

### Computer use and other risk factors of headaches

In recent years, with the popularity of mobile phones and computers, the harm of electromagnetic radiation to the human body has become a serious public health problem, which has already aroused international concern. An increasing number of studies have shown that there is a close relationship between excessive computer use and headache [33–37]. However, although the association between computer use and headache has been established [38–40], only a few studies have diagnosed headache by the latest ICHD-3 guidelines. IT jobs require sitting at a desk and using a computer for many hours a day. Even in their leisure time, the IT staffs are closely in contact with mobile devices such as mobile phones. In our study, more frequent computer use was significantly associated with a greater prevalence of primary headache, especially TTH, which indicated that occupational factors affected the prevalence of headache among the IT population. The multiple logistic regression analysis showed that participants exposed to computers for more than 8 h per day were almost 1.5-fold more likely to suffer from TTH. The multiple logistic regression analysis also indicated that the particular type of job had no effect on the prevalence of migraine and TTH. Our findings suggested that the special environment and the nature of IT work led to a high prevalence rate of tension-type headache in the IT population. The potential mechanism may be as follows. First, a long computer operation time may enhance psychological pressure on IT workers. Anxiety and depression also appear to be followed by TTH. Second, the electromagnetic radiation generated by the computer directly damages the central nervous system, which also contributes to the occurrence of TTH [34–36, 41].

We observed the headache characteristics of primary headache in the IT staff. Photophobia was the most commonly associated symptom (31.80%) in migraine, but in our study, 30% of TTH patients reported photophobia. A large proportion (75.8%) of respondents with any type of headache reported photophobia; this symptom had virtually no discriminative value as a diagnostic criterion, and we could not use it within the framework of ICHD. Photophobia is a technical concept that is not easy to convey to lay participants (even by trained interviewers) [42, 43]. Our eventual solution was to disregard photophobia altogether, and in our view this was necessary: the prevalence estimate for migraine would otherwise have been much higher. In addition, the IT population may be more sensitive to light due to long term work in front of the screen.

### Strengths and limitations of the study

Our study had several strengths. First, this is the first study in Mainland China to assess the potential association between computer use and primary headache. Second, the random cluster sampling method utilized was combined with the high response rate to eliminate selection bias. Furthermore, the diagnosis of headache was based on the latest ICHD-3 guidelines.

The principal limitations of our study first lay in the cross-sectional design. The design did not cover different types of headaches that could have occurred in the same patient, which might require a prospective cohort using headache diaries. In addition, due to the lack of sufficient resources and research capabilities, the interviewers were confronted with numerous difficulties. The survey was also hindered by potential communication barriers between interviewers and participants. Finally, multiple causes involved in headache onset, including anxiety, depression, sleep disturbances, systematic diseases, lifestyle habits and so on. But this study is an epidemiological study mainly aiming to investigate the prevalence of primary headache disorders in informational technology staffs. Due to the huge workload of this study, we didn’t collect as many potential risk factors as possible.

## Conclusion

In a population of information technology staff in Beijing, we found that the one-year prevalence of primary headache was 30.8%. The epidemiological situation is grim. Tension-type headache is the most common headache type in this population. Primary headaches were associated with many factors, among which excessive computer use was the most significant factor contributing to the prevalence of TTH. Although further information is required to enhance our understanding of primary headaches and the working environment among IT populations, proactive preventative strategies should also be developed and evaluated.

It is a well-known fact that prevention is better than cure, but IT staff who spend a long time sitting and staring at screens become the high-risk group for primary headache. We hope to provide a beneficial discussion on the headache situation among IT staff through this paper to reduce the incidence of headaches.

## Data Availability

The dataset supporting the conclusion of this article is available on request to the corresponding author.

## Abbreviations

IT: Informational technology
ICHD: International Classification of Headache Disorders
BMI: Body mass index
TTH: Tension type headache
CIs: Confidence intervals
OR: Odd risk
